# Drowning prevention strategies in rivers: a systematic review 

**Published:** 2022-12

**Authors:** Hamidreza Aghababaeian, Abbas Ostadtaghizadeh, Maryam Kiarsi, Fateme Yazdi, Laden Araghi Ahvazi, Soroush Zohor Soleimani, Shaghayegh Khajavi

**Affiliations:** ^*a*^ Center for Climate Change and Health Research (CCCHR), Dezful University of Medical Sciences, Dezful, Iran.; ^*b*^ Department of Medical Emergencies, Dezful University of Medical Sciences, Dezful, Iran.; ^*c*^ Department of Health in Emergencies and Disasters, School of Public Health, Tehran University of Medical Sciences, Tehran, Iran.; ^*d*^ Center for Climate Change and Health Research (CCCHR), Institute for Environmental Research (IER), Tehran University of Medical Sciences, Tehran, Iran.; ^*e*^ Student Research Committee, Dezful University of Medical Sciences, Dezful, Iran.

## Abstract

**Background:**

Moving towards prevention can be one of the most major factors in helping to reduce drowning deaths in the world. Since rivers are one of the most important and unique places for drowning everywhere, this study was conducted as a systematic review with the aim of determining the strategies to prevent drowning in rivers.

**Methods:**

In this review, WoS, PubMed, Scopus databases, Google Scholar search engine, and some gray literature websites were searched using related keywords including “drowning”, “river”, and “prevention”. Then, the extracted texts were studied by two researchers separately based on the title, abstract and keywords and then the full text. In this study, there was no time limit in the search. If the texts were consistent with the inclusion and exclusion criteria and had the required quality based on the JBI checklist, the results were used in the review study.

**Results:**

Among the 560 searched scientific texts, 70 complete texts were subjected to final evaluation and data extraction after removing duplicate and unrelated items. Out of 251 prevention strategies extracted from the texts, 80 remained after removing similar and duplicate items. Then the extracted strategies were summarized and categorized into 8: identification and research development, exposure reduction, impact reduction, risk understanding, correct policy-making, establishing preventive laws, increasing capacity and capabilities, improving education and safety, and finally improving the environment.

**Conclusions:**

Considering that drowning in rivers is one of the important accidents and emergencies of the health system; extracting, identifying, and classifying strategies to prevent drowning in the world can be a very good guide for researchers, policy-makers, and health planners in order to formulate effective strategies to prevent and reduce the risks of drowning in areas exposed to this risk.

**Keywords:**

Drowning, Emergencies, Strategy, River, Risk Reduction

